# Reintervention Rates After Myomectomy, Endometrial Ablation, and Uterine Artery Embolization for Patients with Uterine Fibroids

**DOI:** 10.1089/jwh.2017.6752

**Published:** 2018-10-12

**Authors:** Matthew R. Davis, Ahmed M. Soliman, Jane Castelli-Haley, Michael C. Snabes, Eric S. Surrey

**Affiliations:** ^1^ Medicus Economics, Milton, Massachusetts.; ^2^ AbbVie, Inc., North Chicago, Illinois.; ^3^ Colorado Center for Reproductive Medicine, Lone Tree, Colorado.

**Keywords:** endometrial ablation, myomectomy, reintervention, uterine artery embolization, uterine fibroids

## Abstract

***Background:*** Women with uterine fibroids (UF) may undergo less invasive procedures than hysterectomy, including myomectomy, endometrial ablation (EA), and uterine artery embolization (UAE); however, long-term need for reintervention is not well characterized. We estimated reintervention rates for 5 years and identified predictors of reintervention.

***Materials and Methods:*** A longitudinal retrospective cohort study was conducted in women in MarketScan^®^ Commercial Claims and Encounters (Truven Health Analytics) aged 18–49 years with UF diagnosis before myomectomy, EA, or UAE from 2008 to 2014. Patients were categorized by initial procedure (index date) and required to have ≥12 months of continuous coverage before and after. Kaplan–Meier analyses and Cox proportional hazard models were used to estimate survival without reintervention and hazard of reintervention for 5 years.

***Results:*** The study included 35,631 women with myomectomy (*n* = 13,804: 8,018 abdominal, 941 hysteroscopic, and 4,845 laparoscopic), EA (*n* = 17,198), and UAE (*n* = 4,629). Myomectomy had the lowest 12-month reintervention rate (4.2%), followed by UAE (7.0%), then EA (12.4%; both *p* < 0.001 relative of myomectomy). Estimates of 5-year reintervention rates were 19% for myomectomy (17%, 28%, and 20% for abdominal, hysteroscopic, and laparoscopic, respectively), 33% for EA, and 24% for UAE. EA and UAE had adjusted hazard ratios of 2.63 (95% confidence interval [CI], 2.44–2.83) and 1.56 (95% CI, 1.42–1.72). Prior anemia, bleeding, pelvic inflammatory disease, and abdominal and pelvic pain increased the hazard of reintervention.

***Conclusion:*** Reintervention rate estimates ranged from 17% to 33% for 5 years after myomectomy, EA, and UAE for patients with UF. Risk of requiring reintervention should be considered during treatment selection.

## Introduction

Uterine leiomyomas (uterine fibroids [UF]) are benign hormonally responsive tumors that form in the wall of the uterus and are common in women aged 30–50 years.^1^ UF are usually asymptomatic but can cause pelvic pain, reproductive problems, and heavy menstrual bleeding that may lead to anemia.^2,3^ For those with severe symptoms, treatment interventions include hysterectomy and other less invasive procedures. Hysterectomy involves removing the uterus, which typically requires hospitalization and can be associated with a lengthy recovery period, and eliminates the ability to carry a pregnancy.^4^

Many women elect to undergo potentially less invasive procedures than hysterectomy, including myomectomy, endometrial ablation (EA), and uterine artery embolization (UAE), in order to conserve their uterus, avoid prolonged recovery times, or potentially retain fertility with myomectomy. These procedures are considered reasonable alternatives to hysterectomy, given their safety profile, patient satisfaction, and quality-of-life scores.^5^ Numerous factors affect which intervention a patient undergoes, including symptom profile, patient preferences regarding recovery period and fertility, insurance, fibroid characteristics (location, number, and size), presence or absence of coexisting pathology, and experience of the physician.^6^ Specific comorbidities such as menstrual disorders and pelvic pain may also influence the treatment decision.^7^

Patients who undergo a more conservative approach than hysterectomy would prefer to avoid additional procedures, but there is limited recent evidence describing long-term reintervention rates with direct comparisons between myomectomy, EA, and UAE, especially in large samples with >1-year of follow-up observation.^8^ Existing evidence primarily comes from clinical trials with only two comparison arms or surveys of patients with variable follow-up periods that often do not adjust for patient characteristics potentially relevant to the treatment decision.

Long-term comparative analysis of patients undergoing procedures for symptoms of UF is an identified research priority; the United States Agency for Healthcare Research and Quality concluded in 2011 that “currently available literature is insufficient to draw conclusions about the relative benefits, harms, or costs of the available choices, making it difficult for patients, providers, payers, and others to select appropriate treatments.”^8^

Several studies have examined reintervention after UAE, including the EMMY, REST, and HOPEFUL trials, which reported cumulative reintervention rates ranging from 23% to 32% for 5–7 years.^9–12^ Reintervention was less common (16%) in a survey of women in the U.K. 5–7 years after UAE and in the U.S. fibroid registry in the 3 years after UAE (14%).^13,14^ Fewer recent studies have been performed for myomectomy and EA. Two U.S. studies in the early 2000s estimated cumulative 5-year reintervention rates of 24% after myomectomy and 22% for hysterectomy after EA.^15,16^ Lastly, a large retrospective analysis of U.K. women who underwent EA found a cumulative 5-year reintervention rate of 17%. However, these studies were generally conducted on a broader population of patients with menorrhagia, which may be due to causes other than UF.^17^

Limited studies have directly compared reintervention rates across treatment options for patients with UF. A higher but not statistically significant number of patients received repeat interventions after abdominal myomectomy (14%) than after UAE (8%) in a study for 50–83 months.^18^ In a long-term U.K. study, reintervention rates were 31% for UAE versus 26% for EA versus 25% for myomectomy for a maximum follow-up of 11 years.^19^ A recent analysis of U.S. electronic health records found that EA had the highest hazard of reintervention, followed by myomectomy, then UAE.^20^ Finally, a large analysis of U.S. commercial claims for women with symptomatic UF found that a significantly higher proportion of women who underwent UAE had reinterventions than those who had a myomectomy (17% vs. 15%); however, this study did not consider EA or myomectomy subtypes.^21^

Depending upon the extent of fibroids, priorities of the patient, and experience of the physician, myomectomy procedures can be abdominal, laparoscopic, or hysteroscopic.^22^ Laparoscopic myomectomy has increased in popularity due to lower rates of hemorrhage, shorter postoperative hospital stays, and less postoperative pain than abdominal myomectomy; however, the comparative risk of recurrence of fibroids and need for reintervention remain uncharacterized.^23–26^

UF-related treatment is associated with substantial healthcare utilization, which results in significant increases in direct and indirect costs.^27,28^ Interventional procedures for UF are expensive, and treatment selection has cost implications for several years.^4,29^ Evidence exists that the need for reintervention following nondefinitive procedures significantly reduces or eliminates the initial cost–benefit relative to hysterectomy.^10^

The objectives of this research were to evaluate rates of reintervention after myomectomy (all known types and divided into the following three subtypes: abdominal, laparoscopic, and hysteroscopic), EA, and UAE for patients with UF in a large commercial claims data set and to identify patient characteristics and comorbidities associated with risk of reintervention.

## Materials and Methods

### Study design and data source

A retrospective analysis of longitudinal patient-level data was conducted using the MarketScan^®^ Commercial Claims and Encounters database (Truven Health Analytics, Ann Arbor, MI). These data provide medical and prescription drug claims for a geographically diverse nationally representative population of >100 million U.S. employees with employer-sponsored insurance and their dependents.^30^ Medical claims include diagnosis and procedure codes that can be used to identify patients with UF and specific interventional procedures. Claims can be linked to enrollment data through unique deidentified enrollee keys to determine periods of continuous coverage. For this study, claims from January 2008 through December 2014 were included. As no patient-identifying information was used in this analysis, institutional review board review was not required.

### Study population

The sample included women aged 18–49 years with a diagnosis for UF, defined as International Classification of Diseases, Ninth Revision, Clinical Modification (ICD-9) code 218.x in any diagnosis field. Subjects must have had at least one initial procedure potentially related to UF (including hysterectomy, myomectomy, EA, UAE, and magnetic resonance-focused ultrasound [MRgFUS]), and a UF diagnosis on the date of their first UF-related procedure (index date) to ensure the treatment was for UF. Patients with an initial procedure that was not accompanied by a UF diagnosis were excluded. Women diagnosed with endometriosis or who underwent an oophorectomy before their index date were excluded, as these could affect the UF treatment selection.

All subjects were required to have ≥12 months of continuous insurance coverage before and after their index date. As such, patients with an index procedure in 2009 through 2013 were included in this study. Patients with a hysterectomy as their earliest UF-related procedure were excluded, as reintervention should not be needed. In addition, patients with MRgFUS as their initial intervention for UF were excluded due to small sample size. Lastly, patients who underwent a myomectomy of an unknown type were excluded due to incomplete information.

### Initial interventions

The study population was divided into cohorts based on the initial interventions for UF-related treatment, identified using procedure codes on inpatient and outpatient medical claims, following the categorization used in prior UF studies.^4,29^ Myomectomy included a composite category of all known myomectomy procedures, as well as subcategories for each of three subtypes: abdominal, hysteroscopic, and laparoscopic. If codes for multiple procedure types were recorded on the same day for a given patient, the intervention was categorized to the more invasive treatment.

### Outcomes

The primary outcome was reintervention, defined as EA, UAE, myomectomy, MRgFUS, or hysterectomy following the initial procedure. The proportion of patients undergoing first reintervention with each procedure type was assessed, as was the time from initial UF-related procedure to reintervention. Reintervention rates were calculated at various lengths of follow-up period among patients with continuous eligibility throughout. To account for delays in claims processing and coding related to follow-up care, a procedure recorded within 2 weeks following a prior procedure of the same type was not considered a separate reintervention, consistent with a previous reintervention study using this database.^31^

### Potential predictors

The primary predictor examined was the initial intervention, including myomectomy (all of known types and separately abdominal, hysteroscopic, and laparoscopic), EA, and UAE. Other potential predictors included patient age, days from earliest observed UF diagnosis to first intervention, U.S. geographic region, year of initial intervention, any history of infertility and UF-related comorbidities present in the 12 months before the index date (assessed using ICD-9 diagnosis codes), along with the Charlson Comorbidity Index (CCI) based on enhanced ICD-9 algorithm.^32^ Specific UF-related comorbidities analyzed were abdominal pain, anemia, endometrial polyp/other disorders of the uterus, heavy menstrual bleeding, obesity, other disorders of menstruation or abnormal bleeding (excluding heavy menstrual bleeding), ovarian cyst, pelvic inflammatory disease, pelvic pain, and urinary problems.

### Statistical analyses

Descriptive statistics assessed differences between cohorts at baseline and at 12 months after the index date. Wilcoxon rank-sum and chi-squared tests were used to compare continuous and categorical variables, respectively, with all comparisons relative to the combined myomectomy of known type cohort. Comparisons across myomectomy subtypes were conducted relative to abdominal myomectomy.

Survival analyses assessed time to reintervention, accounting for censoring due to death, loss of eligibility, or end of the data. Kaplan–Meier analyses estimated the probability of reintervention up to 5 years after the initial procedure. Multivariate Cox proportional hazards regressions estimated the effect of all predictor variables on hazard of reintervention, including procedure type, demographics, and UF-related comorbidities. In all analyses, a two-sided alpha error level of 0.05 was used to indicate statistical significance. All analyses were conducted using SAS 9.4 software (SAS Institute, Inc., Cary, NC).

## Results

### Sample selection and baseline characteristics

**FIG. 1.**
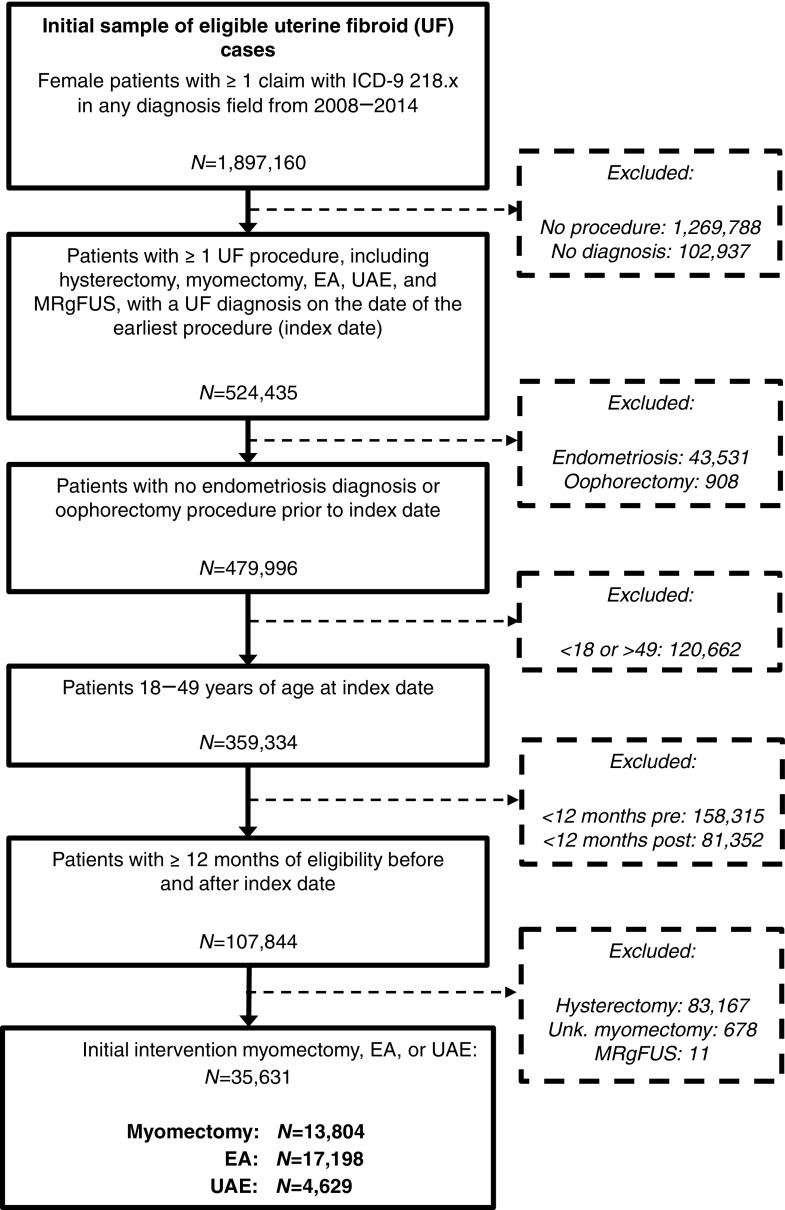
Inclusion and exclusion steps used to construct the analytic sample. Source: Truven MarketScan Commercial Claims and Encounters data for 2008–2014. Patients with hysterectomy initially were excluded (*n* = 83,167), as reintervention would not be observed. Patients with MRgFUS initially were also excluded due to small sample size (*n* = 11). Patients with an initial myomectomy procedure of an unknown type were excluded as well (*n* = 678). EA, endometrial ablation; ICD-9, International Classification of Diseases, Ninth Revision, Clinical Modification code; MRgFUS, magnetic resonance-focused ultrasound; UAE, uterine artery embolization.

A total of 35,631 women met the inclusion criteria and had an initial UF-related procedure with myomectomy (*n* = 13,804 all known types; *n* = 8,018 abdominal, *n* = 4,845 laparoscopic, *n* = 941 hysteroscopic), EA (*n* = 17,198), and UAE (*n* = 4,629) (Fig. 1). The size of the cohorts meeting the same selection criteria who received hysterectomy or MRgFUS as their initial intervention was 83,167 and 11, respectively. There were 678 patients with an initial myomectomy procedure of an unknown type, who were excluded.

The average age was 37.0 years, 42.4 years (*p* < 0.001 relative to myomectomy), and 42.9 years (*p* < 0.001) for patients who received myomectomy, EA, and UAE (Table 1). The average age for patients with the three myomectomy subtypes was 36.5 years for abdominal, 37.0 years for laparoscopic, and 41.0 years for hysteroscopic (both *p* < 0.001 relative to abdominal). Nearly half of the myomectomy and UAE patients were located in the South compared with only 39% of EA patients, whereas more EA patients were located in the North Central and West (both *p* < 0.001). Most women had at least 90 days of gap between their first observed UF diagnosis and their initial myomectomy (57%) or UAE (68%), whereas 69% of patients undergoing EA did so within 90 days of their first observed UF diagnosis (*p* < 0.001 for both). Myomectomy and EA were the more common interventions, representing 38% and 47% of cases in 2009 and 40% and 48% in 2013.

**Table 1.** Baseline Demographics and Comorbidities in the Year Before Initial UF Procedure

	*Myomectomy (of known type)*	*Abdominal myomectomy*	*Hysteroscopic myomectomy*		*Laparoscopic myomectomy*		*EA*		*UAE*	
	n* = 13,804*	n* = 8,018*	n* = 941*	p^a^	n* = 4,845*	p^a^	n* = 17,198*	p^b^	n* = 4,629*	p^b^
*Demographics*										
Mean age (SD)	37.0 (5.6)	36.5 (5.3)	41.0 (6.0)	<0.001	37.0 (5.7)	<0.001	42.4 (5.0)	<0.001	42.9 (4.5)	<0.001
18–29, *n* (%)	1,225 (8.9)	730 (9.1)	39 (4.1)	<0.001	456 (9.4)	0.560	284 (1.9)	<0.001	36 (0.9)	<0.001
30–34, *n* (%)	3,483 (25.2)	2,148 (26.8)	118 (12.5)	<0.001	1,217 (25.1)	0.037	1,053 (6.1)	<0.001	204 (4.4)	<0.001
35–39, *n* (%)	4,546 (32.9)	2,816 (35.1)	179 (19.0)	<0.001	1,551 (32.0)	<0.001	3,037 (17.7)	<0.001	745 (16.1)	<0.001
40–44, *n* (%)	3,164 (22.9)	1,772 (22.1)	295 (31.3)	<0.001	1,097 (22.6)	0.475	5,790 (33.7)	<0.001	1,650 (35.6)	<0.001
45–49, *n* (%)	1,386 (10.0)	552 (6.9)	310 (32.9)	<0.001	524 (10.8)	<0.001	7,034 (40.9)	<0.001	1,994 (43.1)	<0.001
Geographic region, *n* (%)										
Northeast	2,448 (17.7)	1,389 (17.3)	225 (23.9)	<0.001	834 (17.2)	0.873	2,822 (16.4)	0.011	742 (16.0)	0.022
North Central	2,221 (16.0)	1,094 (13.6)	189 (20.1)	<0.001	928 (19.2)	<0.001	4,070 (23.7)	<0.001	879 (19.0)	<0.001
South	6,748 (48.9)	4,176 (52.1)	345 (36.7)	<0.001	2,227 (46.2)	<0.001	6,706 (39.0)	<0.001	2,258 (48.8)	0.333
West	2,176 (15.8)	1,246 (15.5)	173 (18.4)	0.024	757 (15.6)	0.898	3,241 (18.8)	<0.001	637 (13.8)	<0.001
Other/unknown	221 (1.6)	113 (1.4)	9 (1.0)	0.257	99 (2.0)	0.006	359 (2.1)	0.001	113 (2.4)	0.002
Days from earliest diagnosis to first intervention, *n* (%)										
≤90	5,879 (42.6)	2,995 (37.4)	658 (69.9)	<0.001	2,226 (45.9)	<0.001	11,826 (68.8)	<0.001	1,462 (31.6)	<0.001
>90	7,925 (57.4)	5,023 (62.6)	283 (30.1)	<0.001	2,619 (54.1)	<0.001	5,372 (31.2)	<0.001	3,167 (68.4)	<0.001
Year of first UF-related procedure, *n* (%)										
2009	2,282 (16.5)	1,534 (19.1)	156 (16.6)	0.058	592 (12.2)	<0.001	2,794 (16.2)	0.253	909 (19.6)	<0.001
2010	2,597 (18.8)	1,620 (20.2)	171 (18.2)	0.140	806 (16.6)	<0.001	3,204 (18.6)	0.398	963 (20.8)	0.007
2011	3,225 (23.4)	1,847 (23.0)	215 (22.8)	0.897	1,163 (24.0)	0.209	4,103 (23.9)	0.249	1,072 (23.2)	0.837
2012	2,859 (20.7)	1,551 (19.3)	207 (22.0)	0.052	1,101 (22.7)	<0.001	3,641 (21.2)	0.266	847 (18.3)	<0.001
2013	2,841 (20.6)	1,466 (18.3)	192 (20.4)	0.113	1,183 (24.4)	<0.001	3,456 (20.1)	0.639	838 (18.1)	0.001
Days from first intervention to loss of follow-up, mean (SD) [median]	960.7 (462.4) [854]	989.0 (477.5) [888]	982.3 (463.5) [891]	0.944	909.8 (431.6) [796]	<0.001	977.3 (463.0) [880]	<0.001	1,016.5 (488.3) [919]	<0.001
Comorbidities, *n* (%)										
Abdominal pain	3,333 (24.1)	1,949 (24.3)	153 (16.3)	<0.001	1,231 (25.4)	0.161	2,654 (15.4)	<0.001	982 (21.2)	<0.001
Anemia	3,250 (23.5)	2,062 (25.7)	294 (31.2)	<0.001	894 (18.5)	<0.001	4,495 (26.1)	<0.001	1,593 (34.4)	<0.001
Endometrial polyp/other disorders of the uterus	2,339 (16.9)	1,291 (16.1)	230 (24.4)	<0.001	818 (16.9)	0.246	3,916 (22.8)	<0.001	1,018 (22.0)	<0.001
Heavy menstrual bleeding	4,789 (34.7)	2,625 (32.7)	517 (54.9)	<0.001	1,647 (34.0)	0.143	11,942 (69.4)	<0.001	2,448 (52.9)	<0.001
Infertility	2,081 (15.1)	1,118 (13.9)	50 (5.3)	<0.001	913 (18.8)	<0.001	924 (5.4)	<0.001	44 (1.0)	<0.001
Obesity	785 (5.7)	474 (5.9)	60 (6.4)	0.569	251 (5.2)	0.082	1,256 (7.3)	<0.001	310 (6.7)	0.032
Other disorders of menstruation or abnormal bleeding	5,172 (37.5)	2,863 (35.7)	511 (54.3)	<0.001	1,798 (37.1)	0.109	10,689 (62.2)	<0.001	1,733 (37.4)	0.821
Ovarian cyst	2,356 (17.1)	1,201 (15.0)	121 (12.9)	0.082	1,034 (21.6)	<0.001	2,423 (14.1)	<0.001	721 (15.6)	0.062
Pelvic inflammatory disease	2,633 (19.1)	1,494 (18.6)	159 (16.9)	0.194	980 (20.2)	0.026	2,287 (13.3)	<0.001	793 (17.1)	0.008
Pelvic pain	5,421 (39.3)	2,888 (36.0)	252 (26.8)	<0.001	2,281 (47.1)	<0.001	4,648 (27.0)	<0.001	1,514 (32.7)	<0.001
Urinary problems	680 (4.9)	389 (4.9)	36 (3.8)	0.161	255 (5.3)	0.300	484 (2.8)	<0.001	309 (6.7)	<0.001
CCI, mean (SD)	0.22 (0.66)	0.22 (0.66)	0.24 (0.71)	0.424	0.22 (0.66)	0.254	0.28 (0.76)	<0.001	0.27 (0.71)	<0.001

Source: Truven MarketScan Commercial Claims and Encounters data for 2008–2014.

Demographics assessed at the time of initial intervention. Comorbidities evaluated in the 12 months before the first UF-related procedure.

^a^ Wilcoxon rank-sum tests and chi-squared tests were used to compare continuous and categorical variables, respectively.

^b^ All comparisons are relative to the myomectomy cohort.

CCI, Charlson Comorbidity Index; EA, endometrial ablation; UAE, uterine artery embolization; UF, uterine fibroids.

The CCI indicates that EA patients (mean, 0.28; *p* < 0.001) and UAE patients (mean, 0.27; *p* < 0.001) had a higher comorbidity burden than myomectomy patients (mean, 0.22). There was no significant difference in CCI between the three myomectomy subtypes (mean, 0.22, 0.24, and 0.22 for abdominal, hysteroscopic, and laparoscopic, respectively). For comorbidities associated with UF, EA patients had higher prevalence of bleeding abnormalities (e.g., the proportion of patients with heavy menstrual bleeding was 35% for myomectomy, 69% [*p* < 0.001] for EA, and 53% [*p* < 0.001] for UAE, and the proportion with other disorders of menstruation or abnormal bleeding was 38% for myomectomy, 62% [*p* < 0.001] for EA, and 37% [*p* < 0.001] for UAE).

A higher proportion of myomectomy patients had pain (e.g., the proportion of patients with pelvic pain was 39% for myomectomy, 27% [*p* < 0.001] for EA, and 33% [*p* < 0.001] for UAE, and the proportion with abdominal pain was 24% for myomectomy, 15% [*p* < 0.001] for EA, and 21% [*p* < 0.001] for UAE) at baseline. A higher proportion of UAE patients had anemia (34%) relative to EA (26%) and myomectomy patients (24%; *p* < 0.001). Infertility was most common among myomectomy patients at 15% relative to 5% of EA and 1% of UAE patients (*p* < 0.001).

Among the three myomectomy subtypes, patients who received laparoscopic myomectomy had the highest proportion of patients who reported pelvic pain compared with patients who received hysteroscopic or abdominal myomectomies (47% compared with 27% and 36%, respectively). There were also significant differences across myomectomy procedure types with regard to a history of infertility; 19% of women who received laparoscopic myomectomies had a history of infertility compared with 5% of women who received hysteroscopic and 14% of women who received abdominal myomectomies.

### Reintervention rates

Patients treated with EA had the highest observed rate of reintervention in each follow-up period, followed by UAE, then myomectomy. At 1 year after the index procedure, 4%, 12% (*p* < 0.001), and 7% (*p* < 0.001) of patients who received myomectomy, EA, and UAE, respectively, had a reintervention (Table 2).

**Table 2.** Reintervention in the First Year After Initial UF Procedure

	*Myomectomy (of known type)*	*Abdominal myomectomy*	*Hysteroscopic myomectomy*		*Laparoscopic myomectomy*		*EA*		*UAE*	
	n* = 13,804*	n* = 8,018*	n* = 941*	p^a^	n* = 4,845*	p^a^	n* = 17,198*	p^b^	n* = 4,629*	p^b^
Reintervention in the first year after initial procedure										
Reintervention, *n* (%)^c^	560 (4.1)	244 (3.0)	113 (12)	<0.0001	203 (4.2)	0.0006	2,139 (12.4)	<0.001	324 (7.0)	<0.001
Days to reintervention, mean (SD)	149 (108.6)	172 (111.2)	117 (101.1)	<0.0001	138 (103.8)	0.0011	158 (107.4)	0.017	175 (115.9)	<0.001
Cumulative proportion of patients undergoing reintervention, *n* (%)										
1 month	89 (1)	25 (0)	24 (3)	<0.0001	40 (1)	<0.0001	235 (1.4)	<0.001	54 (1.2)	<0.001
3 months	210 (2)	77 (1)	56 (6)	<0.0001	77 (2)	0.0015	745 (4.3)	<0.001	105 (2.3)	0.003
6 months	360 (3)	140 (2)	87 (9)	<0.0001	133 (3)	0.0001	1,273 (7.4)	<0.001	162 (3.5)	0.007
9 months	463 (3)	184 (2)	100 (11)	<0.0001	179 (4)	<0.0001	1,716 (10.0)	<0.001	239 (5.2)	<0.001
12 months	560 (4)	244 (3)	113 (12)	<0.0001	203 (4)	0.0006	2,139 (12.4)	<0.001	324 (7.0)	<0.001
No. of reinterventions, *n* (%)										
0	13,244 (95.9)	7,774 (97.0)	828 (88.0)	<0.0001	4,642 (95.8)	0.0006	15,059 (87.6)	<0.001	4,305 (93.0)	<0.001
1	545 (3.9)	238 (3.0)	109 (11.6)	<0.0001	198 (4.1)	0.0007	2,035 (11.8)	<0.001	303 (6.5)	<0.001
2	13 (0.1)	5 (0.1)	4 (0.4)	<0.0001	4 (0.1)	0.6746	97 (0.6)	<0.001	13 (0.3)	0.015
>2	2 (0.0)	1 (0.0)	0 (0.0)	0.7319	1 (0.0)	0.7188	7 (0.0)	0.157	8 (0.2)	<0.001
First reintervention procedure, *n* (% of patients with reintervention)										
Hysterectomy	209 (37)	79 (32)	52 (46)	<0.0001	78 (38)	0.0018	1,383 (65)	<0.001	186 (57)	<0.001
Myomectomy	110 (20)	52 (21)	10 (9)	0.1471	48 (24)	0.0323	196 (9)	0.004	43 (13)	0.460
Abdominal	47 (8)	32 (13)	3 (3)	0.8253	12 (6)	0.1615	88 (4)	0.0056	18 (6)	0.5570
Hysteroscopic	9 (2)	4 (2)	3 (3)	0.0030	2 (1)	0.8341	30 (1)	0.0031	13 (4)	0.0002
Laparoscopic	49 (9)	102 (42)	4 (4)	0.0379	33 (16)	<0.0001	65 (3)	0.4281	7 (2)	0.0357
Unknown	5 (1)	11 (5)	0 (0)	0.5127	1 (0)	0.4195	13 (1)	0.1079	5 (2)	0.0616
EA	244 (40)	102 (42)	47 (42)	<0.0001	75 (37)	0.1932	498 (23)	<0.001	56 (17)	0.034
UAE	17 (3)	11 (5)	4 (4)	0.0410	2 (1)	0.0972	62 (3)	<0.001	39 (12)	<0.001
MRgFUS	0 (0)	0 (0)	0 (0)	1.0000	0 (0)	1.000	0 (0)	1.000	0 (0)	1.000

Source: Truven MarketScan Commercial Claims and Encounters data for 2008–2014.

Outcomes evaluated in the 12 months after the initial UF-related procedure for each patient.

^a^ Wilcoxon rank-sum tests and chi-squared tests were used to compare continuous and categorical variables, respectively.

^b^ All comparisons are relative to the myomectomy cohort.

^c^ Based on unique service dates of claims with a procedure code for UF-related intervention. To account for delays in claims processing and coding related to follow-up care, a procedure within 2 weeks of the prior procedure, and of the same procedure type was not considered a reintervention.

MRgFUS, magnetic resonance-focused ultrasound.

Among patients with a reintervention within the first year, the average time between the initial intervention and reintervention was shortest for myomectomy (149 days), followed by EA (158 days; *p* = 0.017) and UAE (175 days; *p* < 0.001). For these patients, roughly 60% of patients with EA and UAE initially progressed to hysterectomies (both *p* < 0.001 relative to 37% for myomectomy). Reintervention with EA was the next most common, with 40% of myomectomy, 23% of EA (*p* < 0.001), and 17% of UAE (*p* = 0.047) reintervention patients. Less than 1% of patients in each cohort had multiple reinterventions in the first year.

Among myomectomy subtypes, patients who received hysteroscopic myomectomies had the highest rate of reinterventions within the first year (12%) than patients who received abdominal (3%) and laparoscopic myomectomies (4%).

**FIG. 2.**
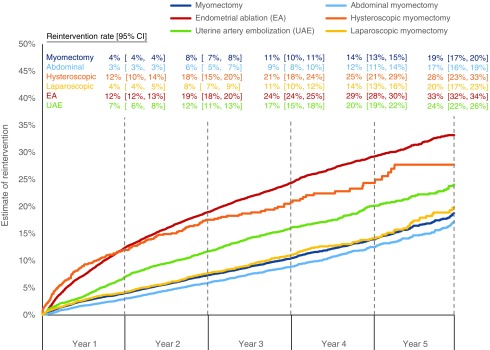
Kaplan–Meier estimates of reintervention rates by initial procedure type. Source: Truven MarketScan Commercial Claims and Encounters data for 2008–2014. Estimates were produced using a nonparametric survival analysis of time to first reintervention with patients censored based on loss of eligibility. CI, confidence interval.

Kaplan–Meier estimates of reintervention at 1 and 5 years after an initial procedure increased from 4% (CI, 4%–4%) to 19% (CI, 17%–20%) for myomectomy, from 12% (CI, 12%–13%) to 33% (CI, 32%–34%) for EA, and from 7% (CI, 6%–8%) to 24% (CI, 22%–26%) for UAE (Fig. 2). For myomectomy subtypes, Kaplan–Meier estimates of reintervention at 1 and 5 years after an initial procedure increased from 3% (CI, 3%–3%) to 17% (CI, 16%–19%) for abdominal, from 12% (CI, 10%–14%) to 28% (CI, 23%–33%) for hysteroscopic, and from 4% (CI, 4%–5%) to 20% (CI, 17%–23%) for laparoscopic myomectomies. Survival without reintervention for both EA and UAE was significantly shorter than myomectomy based on a log-rank test (*p* < 0.001).

### Predictors of reintervention

A multivariate Cox proportional hazards model controlling for procedure type, demographics, and baseline comorbidities was used to produce hazard ratio (HR) estimates, representing the ratio of the hazard of reintervention relative to a reference group. EA and UAE had a higher hazard of reintervention than myomectomy, with HRs of 2.63 (CI, 2.44–2.83) and 1.56 (CI, 1.42–1.72), respectively (Table 3). Compared with women aged 18–29 years, patients who were aged 30–34, 35–39, and 40–44 years at their first UF procedure had an increased hazard of reintervention of 1.30 (CI, 1.08–1.56), 1.38 (CI, 1.16–1.64), and 1.35 (CI, 1.13–1.60), respectively.

**Table 3.** Risk of Reintervention Associated with the Initial Intervention and Other Factors

	*Model of any reintervention with myomectomy of known type*		*Model of any reintervention with myomectomy subtypes*	
n* = 35,631*	*HR*	*95% CI*	*HR*	*95% CI*
	*Initial procedure (reference: myomectomy of known type)*		*(Reference: abdominal myomectomy)*	
Hysteroscopic myomectomy	—	—	2.79	2.36–3.29
Laparoscopic myomectomy	—	—	1.25	1.11–1.41
EA	2.63	2.44–2.83	3.23	2.94–3.54
UAE	1.56	1.42–1.72	1.89	1.69–2.11
Demographics				
Age group (reference: 18–29)				
30–34	1.30	1.08–1.56	1.29	1.08–1.55
35–39	1.38	1.16–1.64	1.35	1.14–1.61
40–44	1.35	1.13–1.60	1.30	1.09–1.54
45–49	1.04	0.87–1.23	0.99	0.83–1.18
Geographic region (reference: South)				
Northeast	0.98	0.91–1.06	0.97	0.90–1.05
North Central	1.03	0.97–1.11	1.03	0.96–1.10
West	0.93	0.86–1.00	0.92	0.85–0.99
Other/unknown	1.01	0.83–1.22	1.01	0.83–1.23
Year of first UF-related procedure (reference: 2009)				
2010	0.94	0.87–1.02	0.94	0.87–1.02
2011	0.97	0.90–1.05	0.97	0.90–1.05
2012	0.99	0.91–1.08	0.98	0.90–1.07
2013	0.95	0.87–1.05	0.94	0.86–1.04
>90 days from initial UF diagnosis to first intervention (reference: ≤90)	1.08	1.02–1.14	1.12	1.06–1.18
Comorbidities				
Abdominal pain	1.10	1.03–1.18	1.11	1.04–1.18
Anemia	1.29	1.22–1.36	1.29	1.22–1.36
Endometrial polyp/other disorders of the uterus	0.95	0.89–1.02	0.95	0.89–1.01
Heavy menstrual bleeding	1.15	1.09–1.22	1.14	1.07–1.21
Infertility	1.05	0.95–1.17	1.06	0.96–1.18
Obesity	0.98	0.88–1.09	0.98	0.88–1.09
Other disorders of menstruation or abnormal bleeding	1.07	1.01–1.13	1.06	1.00–1.11
Ovarian cyst	0.99	0.92–1.07	0.99	0.92–1.07
Pelvic inflammatory disease	1.09	1.01–1.13	1.08	1.01–1.16
Pelvic pain	1.09	1.03–1.15	1.09	1.03–1.16
Urinary problems	1.10	0.97–1.26	1.10	0.97–1.26
CCI	1.01	0.97–1.04	1.01	0.97–1.04

Source: Truven MarketScan Commercial Claims and Encounters data for 2008–2014.

Estimates were from a Cox proportional hazards model of reintervention after controlling for the initial intervention type and other characteristics assessed before the initial UF intervention. HRs >1 indicate increased hazard of reintervention relative to the reference group.

CI, confidence interval; HR, hazard ratio.

Having the following comorbidities during the baseline period significantly raised the hazard of reintervention (no comorbidities analyzed significantly lowered the hazard): abdominal pain (HR, 1.10; CI, 1.03–1.18), anemia (HR, 1.29; CI, 1.22–1.36), heavy menstrual bleeding (HR, 1.15; CI, 1.09–1.22), other disorders of menstruation or abnormal bleeding (HR, 1.07; CI, 1.01–1.13), pelvic inflammatory disease (HR, 1.09; CI, 1.01–1.13), and pelvic pain (HR, 1.09; CI, 1.03–1.15). Relative to abdominal myomectomy, all other intervention types had a significantly higher hazard of reintervention (2.79 [CI, 2.36–3.29] for hysteroscopic myomectomy; 1.25 [CI, 1.11–1.41] for laparoscopic myomectomy; 3.23 [CI, 2.94–3.54] for EA; 1.89 [CI, 1.69–2.11] for UAE).

## Discussion

Reintervention comprises a significant burden for UF patients, and variation in reintervention rates across procedures should be an important consideration in treatment selection. The goal of this research is to inform patients, providers, and insurers of average rates of reintervention after myomectomy (of known type combined and by subtype, exclusive of unknown procedures), EA, and UAE for symptomatic UF patients. This builds on prior research, which mostly relied on surveys with limited comparison between treatments in the same population.

This study of a large nationally representative commercially insured population found that 4%–12% of UF patients had a reintervention within the first year. EA is associated with the highest rate of reintervention, with Kaplan–Meier estimates indicating a 33% likelihood of reintervention for the 5 years after the initial procedure, followed by UAE at 24% and myomectomy at 19% (all differences statistically significant). Of the three myomectomy subtypes, abdominal had the lowest rate of reintervention, followed by laparoscopic then hysteroscopic. Age- and UF-related comorbidities significantly affected the hazard of reintervention.

Many factors should be considered before making a choice of therapy, including patient preferences and attributes of specific modalities. Patients who wish to maintain fertility may opt for myomectomy instead of EA. Conversely, patients who experience heavy bleeding may be more likely to undergo EA or UAE. Myomectomy has been associated with the longest recuperation period, and UAE with the shortest.^33^ Absenteeism and disability days follow a similar trend.^4^ EA has been shown to be the least expensive, in terms of payments from the insurer to providers, and UAE the most expensive.^4,34^ Quality-of-life differences reported to date in the year after intervention have not been appreciably different, although this is an area of active research.^33,35,36^ Rate of reintervention is another factor that patients and providers could consider when selecting treatment.

The reintervention rate estimates for each procedure type are generally consistent with prior research. A similar claims-based study in the U.S. evaluating women treated for uterine leiomyoma also reported a higher cumulative incidence of reintervention for UAE than for myomectomy (17% vs. 15%) after a mean follow-up of 3.4 years.^21^ Although the overall reintervention rates were lower than those found in this study, they were higher for patients with 5 years of data (23% vs. 27%), suggesting differential follow-up could have affected the results. In addition, their study did not consider EA as a reintervention and used a broader definition of UF while also requiring bulk symptoms that could have influenced the treatment selection.

Another study in the U.K. found that cumulative incidences of reintervention after myomectomy (25%) and EA (26%) were lower than after UAE (31%) over a maximum of 11 years, and estimated 12-month reintervention rates of 10%, 13%, and 14% for myomectomy, EA, and UAE, respectively, relative to 4%, 12%, and 7% in our study.^19^ Discrepancies between the results could potentially be due to the longer follow-up period in their study and differences in the U.K. patient population and treatment practices. As the authors note, increased use of UAE and EA in the UK during the study period may have impacted reintervention rates.^19^

For UAE, the 5-year reintervention rate falls within the range reported from the EMMY, REST, and HOPEFUL trials.^9–12^ The myomectomy reintervention rate estimate is only slightly lower than that reported in an earlier retrospective analysis of patients who underwent myomectomy from 1993 through 2002.^15^ The reintervention rate estimates by myomectomy subtypes are also consistent with previous research that has shown similar recurrence rates between abdominal and laparoscopic myomectomies, but higher recurrence rates for hysteroscopic myomectomies.^23,37–39^ This phenomenon is not surprising in that patients who undergo hysteroscopic myomectomy may have other fibroids that are not addressed during the initial surgery and are more likely to experience incomplete fibroid resection. Although the reintervention rate for EA is higher than in prior studies, those studies included a broader population of women with menorrhagia and noted that the subset with UF had increased risk of reintervention.^17^ Similar to the results of this study, several studies have reported higher rates of reintervention after UAE relative to myomectomy.^11,33,40^

Only a single other U.S. study has directly compared myomectomy, EA, and UAE, finding reintervention hazard rates of 0.67 (CI, 0.54–0.84) for myomectomy and 0.55 (CI, 0.39–0.79) for UAE relative to EA.^20^ Approximating their model specification, we find a similar estimate for UAE (HR, 0.61; CI, 0.56–0.66), but a lower estimate for myomectomy (HR, 0.39; CI, 0.36–0.42). This discrepancy could be due to different primary insurers or because they include a covariate for race, which we did not have access to in our research. In addition, their study relied on older data (2005–2011) and a smaller population, which may have different usage of specific myomectomy procedures associated with higher rates of reintervention, such as hysteroscopic myomectomy.

This study uses a large recent insurance claims database to compare nonhysterectomy options for treating patients with symptomatic UF. Prior estimates of reintervention rates were primarily from surveys or clinical trials that often relied on small samples and variable follow-up periods due to patient attrition. The majority compared at most two procedures, with limited adjustment for risk factors or treatment history. Using an insurance claims database allows for a direct comparison of multiple procedure types after adjusting for patient characteristics and health history. Administrative claims data provide certain strengths. For example, they typically allow for analysis of larger samples than are available in clinical trials and contain extensive longitudinal information. In addition, claims data more accurately reflect real-world patterns of medical resource use not affected by monitoring and enrollment biases in clinical trials. Furthermore, relying on claims data avoids biases inherent in patient-reported outcomes.

A limitation of claims data is that disease severity and other clinical variables are absent from the data; however, in this case, patients reported herein had sufficient symptoms to require an interventional procedure. Although a UF diagnosis was required, it is possible that patients underwent the interventions examined primarily for conditions or symptoms unrelated to UF. Even if the procedure was for UF, we could not determine with certainty whether UF-related pain or bleeding ultimately led to the intervention, or whether treatment decisions were influenced by other comorbidities. However, a sensitivity analysis excluding patients with a history of pelvic inflammatory disease or prior ovarian cystectomy showed similar results.

Furthermore, the data did not allow for differentiation between unexpected reinterventions and planned completion procedures. In addition, the data set is restricted to commercially insured patients, so generalizability to a broader population (such as those with government-provided insurance) may be limited. The inclusion criteria did not allow generalization of results to women with endometriosis or prior oophorectomy, nor to women who had UF-related procedures without a concurrent UF diagnosis recorded. Patients with an initial intervention of myomectomy of an unknown type were also excluded from the study; however, results were largely unchanged in a sensitivity analysis after including these patients.

It is possible that differences in outcomes may be attributable to factors unobservable in claims data, in addition to the observed treatment decisions, such as desire to maintain fertility, clinical characteristics (e.g., number, location, and size of fibroids), health plan, and provider specialty. EA may not be directly comparable with UAE and myomectomy because it specifically targets menorrhagia associated with UF, instead of the fibroids themselves. Presence of heavy bleeding is controlled for in multivariate analyses along with other symptoms of UF, such as pelvic pain; however, claims data are limited in determining the precise reason for treatment selection and/or reintervention. Health history and UF treatments received before entering the data were not observed, and the earliest observed intervention in the data may have not been the actual first intervention for a given patient.

To allow for delays in claims processing, procedures occurring within 2 weeks of a prior procedure of the same type were not considered reintervention, which could cause an underestimation of the true reintervention rate. Finally, with the exception of the myomectomy subtypes examined, this study used broad categorizations of UF treatments and did not estimate variations in reintervention rates by more specific approaches used (such as resectoscopic or nonresectoscopic EA), which could be a subject of future research.

## Conclusions

This study demonstrates that the need for reintervention in the treatment of patients with UF is common and differs across the interventional procedures to manage patients with UF. Although selection of a particular intervention should be specific to the patient, provider, and clinical profile, the risk of requiring reintervention should be an important consideration when determining the appropriate treatment approach for patients with UF, given the cost and patient burden of additional interventions. The interventions examined in this study differ in important dimensions beyond rates of reintervention and should not be viewed as interchangeable or perfectly substitutable. Additional research should further examine the implications of treatment selection by assessing the risk of procedural complications, the need for additional medical treatment for UF, and the cost consequences of reintervention.
